# Overexpression of the 16‐kDa α‐amylase/trypsin inhibitor RAG2 improves grain yield and quality of rice

**DOI:** 10.1111/pbi.12654

**Published:** 2016-11-22

**Authors:** Wei Zhou, Xin Wang, Dan Zhou, Yidan Ouyang, Jialing Yao

**Affiliations:** ^1^ College of Life Science and TechnologyHuazhong Agricultural UniversityWuhanChina; ^2^ National Key Laboratory of Crop Genetic Improvement and National Center of Plant Gene Research (Wuhan)Huazhong Agricultural UniversityWuhanChina

**Keywords:** *Oryza sativa*, *RAG2*, seed storage proteins, grain weight, grain quality

## Abstract

Increasing grain yield and improving grain quality are two important goals for rice breeding. A better understanding of the factors that contribute to the overall grain quantity and nutritional quality of rice will lay the foundation for developing new breeding strategies. RAG2 is a member of 14‐to‐16‐kDa α‐amylase/trypsin inhibitors in rice, which belong to the albumin of seed storage proteins. We found that *RAG2* was specifically expressed in ripening seed and its transcription peak was between 14 and 21 days after flowering. Grain size and 1000‐grain weight were obviously increased in *RAG2*‐overexpressed lines compared with wild type, and grain size was reduced in *RAG2*‐suppressed lines. In addition, the major storage substances of the seeds differed significantly in *RAG2*‐overexpressed and *RAG2*‐suppressed lines compared to wild type. The protein content and amount of total lipids were increased and decreased, respectively, in the seeds of *RAG2*‐overexpressed and *RAG2*‐suppressed lines. Overexpression of *RAG2* significantly increased grain size and improved grain quality and yield simultaneously. These results imply that *RAG2* might play an important role in regulating grain weight and seed quality of rice. The functional characterization of rice *RAG2* facilitates a further understanding of the mechanisms involved in grain size and seed quality and may be helpful in improving grain yield and quantity in cereal crops.

## Introduction

Global food security is challenged by the convergence of multiple factors, including continuously growing population, reduced arable land, demand for biofuel production and global climate change (Zhang *et al*., 2008). Rice (*Oryza sativa*) is the most widely consumed staple food crop that feeds more than half of the world's human population. In addition, with the improved quality of life, people are increasingly concerned about the quality of food. Accordingly, increasing grain yield and improving grain quality of food crops are two important goals of basic and applied science research in plants (Sakamoto and Matsuoka, 2004; Zhang, 2007).

Grain yield of rice is determined by three major factors, including the number of tillers per plant, the number of filled grains per panicle and 1000‐grain weight (Sakamoto and Matsuoka, 2008). Grain weight is primarily determined by grain size (volume) and the degree of grain filling (plumpness) (Sakamoto and Matsuoka, 2008; Xing and Zhang, 2010). Grain size or shape is also an important quality trait of rice grains because of varied consumer preferences in different geographical areas (Zuo and Li, 2014). Grain size is specified by its three dimensions: grain length, grain width and grain thickness. As a quantitative trait, grain size is predominantly and tightly controlled by genetic factors. In recent years, several important genes that control the grain size in rice have been identified. *GW2* encodes a previously unidentified RING‐type protein with E3 ubiquitin ligase activity and affects spikelet hull width by regulating cell numbers (Song *et al*., 2007). *qGL3/qGL3.1* encodes a novel putative serine/threonine protein phosphatase. The reduced phosphatase activity may increase the cell number of the outer glume, resulting in longer grains (Zhang *et al*., 2012). *GS3* is a major quantitative trait locus (QTL) that modulates grain length by controlling the number of the cell in the upper epidermis of the glume (Fan *et al*., 2006). *GS5* encodes a putative serine carboxypeptidase and functions as a positive regulator of grain width (Li *et al*., 2011; Tan *et al*., 2000). *GW5* encodes a novel nuclear protein that physically interacts with polyubiquitin. Within the ubiquitin–proteasome pathway, *GW5* regulates cell division in the outer glumes during seed development (Wan *et al*., 2008). *GW8* encodes a transcription factor OsSPL16 and positively regulates grain width and grain weight via the promotion of cell proliferation (Wang *et al*., 2012).

Grain weight is also affected by grain plumpness, which is mainly controlled by the accumulation of storage substances, such as starch, proteins and lipids. *FLO2* plays a pivotal regulatory role in grain size and starch quality by affecting the accumulation of these substances in the rice endosperm (She *et al*., 2010). The mutation of rice starch regulator1 (*RSR1*) produces a larger seed and increases seed mass and yield (Fu and Xue, 2010). Mutation of *OsSUT2*, which encodes a tonoplast‐localized sucrose transporter, leads to a growth retardation phenotype with a notable reduction in grain weight (Eom *et al*., 2011). Maize (*Zea mays*) *opaque2* (*o2*) encodes a basic Leu zipper transcription factor. Mutations of *opaque2* result in a severe reduction in 22‐kDa α‐zein accumulation in seeds and grain filling (Prioul *et al*., 2008).

Seed storage proteins (SSPs) are one of the main factors determining the nutritional quality of rice. Based on their solubility properties, the SSPs of rice are classified into glutelin, prolamin, albumin and globulin (Yang *et al*., 2013). Glutelins are major SSPs of rice, accounting for 60%–80% by weight of the total seed protein content, and are encoded by 15 genes copies in the rice genome. Glutelins are classified into four subfamilies (GluA, GluB, GluC and GluD) based on amino acid sequence similarity (Kawakatsu *et al*., 2008). The prolamins make up 20%–30% of the seed protein and are encoded by a multigene family of 34 gene copies in three groups defined by their relative molecular weights (Saito *et al*., 2012; Xu and Messing, 2009). Rice seed albumin is classified as the water‐soluble fraction, which comprises about 5% of the total seed protein (Mawal *et al*., 1987). The major albumin exhibits heterogeneity in its molecular size (14–16 kDa) and isoelectric point (pI 6–8), and it shows immunological cross‐reactivity (Tsukasa Matsuda *et al*., 1991). The promoters of several *GLUTELIN* genes (*GluA‐1*,* GluA‐2*,* GluA‐3* and *GluB‐3*) are detected in the peripheral region of the endosperm, whereas *GluB‐5*,* GluC* and *GluD* are active in various regions of the starch endosperm (Komatsu and Hirano, 1992; Lee *et al*., 2015). *NRP33* encoding a 13‐kDa prolamin polypeptide has been cloned (Sha *et al*., 1996). Previous studies revealed that reducing the expression level of rice SSP‐related genes, such as *GluA*,* GluB*,* RP10* (rice prolamins oryzein10) and *RP16* (rice prolamins oryzein16), leads to changes in SSP content (Kawakatsu *et al*., 2010; Xu and Messing, 2009). Increasing the nutrient quality of rice through changing the SSP content is a critical goal in rice breeding. Expression of a soybean β‐globin gene in transgenic rice led to a 4% increase in total protein content in transgenic seeds (Zheng *et al*., 1995). Expression of a soybean glycinin gene in transgenic rice resulted in a significant improvement of glutelin storage in transgenic seed (Katsube *et al*., 1999). An interesting feature of the *Lgc1* lines is that glutelin‐content mutants (*Lgc1*) are used to produce ‘super low‐protein rice’ for patients with kidney disease (Kusaba *et al*., 2003).

Fatty acid (FA) content is a quality‐related factor affecting rice appearance, eating quality and storage (Kim *et al*., 2015). FA deficiency is known to have a major impact on human health in developing countries. Down‐regulated expression of *OsLTPL36* led to decreased FA content and reduced seed quality of rice (Wang *et al*., 2015). Overexpression of *Arabidopsis thaliana SFD1/GLY1*, a gene encoding plastid‐localized glycerol‐3‐phosphate dehydrogenase, increased seed lipid content in transgenic rice (Singh *et al*., 2016). These studies also contribute to rice molecular breeding focusing on improving grain quality.

The albumin gene family (*RAGs*) contains five genes, which are highly expressed at the seed maturation stage (Alvarez *et al*., 1995). Few studies of *RAG* genes have been reported. The expression of *RAG1* is trans‐activated by RPBF (rice prolamin box binding factor) (Kawakatsu *et al*., 2009). The *RAG2* gene was obtained by screening a rice genomic library with a probe clone RA17 (coding the 16‐kDa protein) (Adachi *et al*., 1993), and it is a member of the 14‐to‐16‐kDa α‐amylase/trypsin inhibitors of rice and contains 10 cysteine residues (Adachi *et al*., 1993; Alvarez *et al*., 1995). RAG2 has been reported to be a major allergen in rice (Kurokawa *et al*., 2014; Selgrade *et al*., 2009). *RAG2* is specifically expressed in ripening seed (Kurokawa *et al*., 2014), but the biological functions of rice *RAG2* remain elusive.

In this study, we examined the expression pattern of *RAG2* using transcriptional profiling and *in situ* hybridization tests. The results show that *RAG2* is specifically expressed in the developing seeds, with the highest expression level in seeds of 14–21 DAP. Overexpression and RNAi were carried out to increase and reduce the expression of *RAG2*, respectively. Grain size and 1000‐grain weight were obviously increased in *RAG2*‐OX lines compared to wild type (WT). Furthermore, three major storage substances of the seeds were changed in different ways in *RAG2*‐OX and RNAi lines. The content of proteins and lipids was increased significantly in *RAG2*‐OX lines. These results imply that RAG2, a seed‐specific expression protein, may play an important role in regulating storage substances and thereby affect seed plumpness and ultimately control the grain yield and quality of rice. The discovery of *RAG2* may facilitate increased seed production and improvement of seed quality, and it can also be effectively applied to crop breeding programs.

## Results

### Structural and sequence analysis of *RAG2*

**Figure 1:**
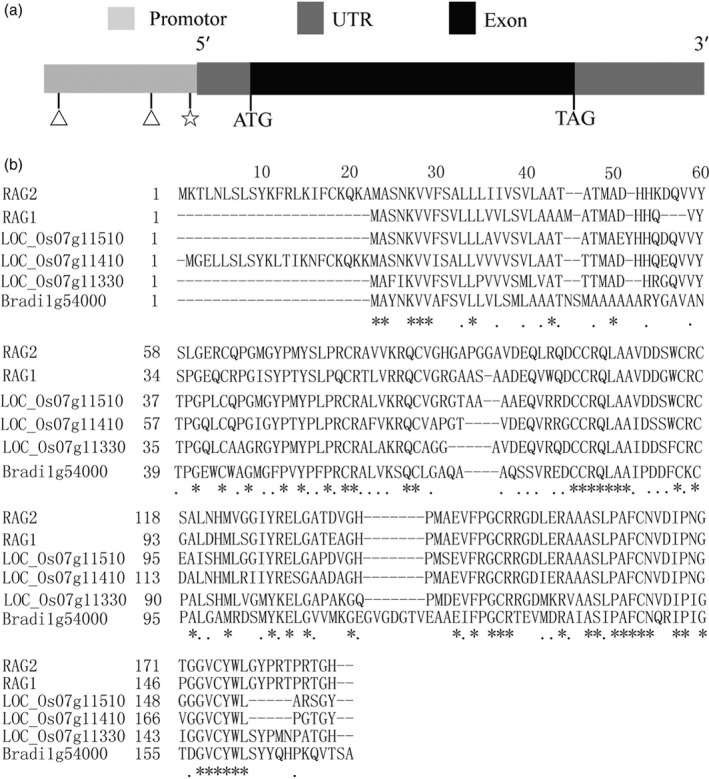
Structural and sequence analysis of *RAG2*. (a) A schematic representation of the exon and intron organization of *RAG2*. *RAG2* consists of one exon (black box) with an 82‐bp 5′UTR (grey box) and a 200‐bp 3′UTR (grey box). Two ATGCAAAA (triangle, −1028 bp, −252 bp) and one CTTTAGTCTT (pentagon, −21 bp) cis‐element in *RAG2* promoter region. (b) Protein sequence alignment of RAG2 with RAG1, LOC_Os07g11510, LOC_Os07g11410, LOC_Os07g11330 and Bradi1g54000. Residues marked with asterisks and dots are highly conserved and semiconserved, respectively. A dash ‘–’ denotes a gap in the alignment.

RAG2 is a member of 14‐to‐16‐kDa α‐amylase/trypsin inhibitors in rice, which are albumin proteins homologous to α‐amylase/trypsin inhibitor family proteins. The genomic DNA of *RAG2* is 783 nucleotides with one exon (http://rice.plantbiology.msu.edu/cgi-bin/ORF_infopage.cgi?orf=LOC_Os07g11380). The cDNA contained a 498‐bp open reading frame (excluding the stop codon), which encoded a 166‐amino acid protein with a theoretical molecular mass of about 16 kDa and pI of 5.4 (AK107328) (Figure 1a). *RAG2* displays high homology with another cDNA clone, *RAG1* (Figure 1b). In addition, two 8‐bp direct repeat units (ATGCAAAA) existed in the *RAG2* promoter (Figure 1a). This consensus sequence ATGCAAAA, which reminisces the heptamer sequence TGCAAAA, was identified in rice glutelin genes (Okita *et al*., 1989) and the ‐300‐bp element in cereal genes (Colot *et al*., 1987; Maier *et al*., 1987). CTTTCGTGTA has been identified as the recognition site of a DNA‐binding protein, particularly in the glutelin promoter. This site is similar to the sequence CTTTAGTCTT in the *RAG2* promoter region (Figure 1a). The transcriptional initiation site of RAG2 gene might be the same as that of RAG1 gene because the nucleotide sequence around the comparable region is similar (Adachi *et al*., 1993). Previous studies found that *RAG2* was localized mainly in protein bodies II (PB‐II) of the endosperm cells (Kurokawa *et al*., 2014). The protein annotation on the Pfam website indicated that *RAG2* belonged to a protease inhibitor/seed storage/LTP family (CL0482).

### Specific high expression of *RAG2* in developing rice seeds

**Figure 2:**
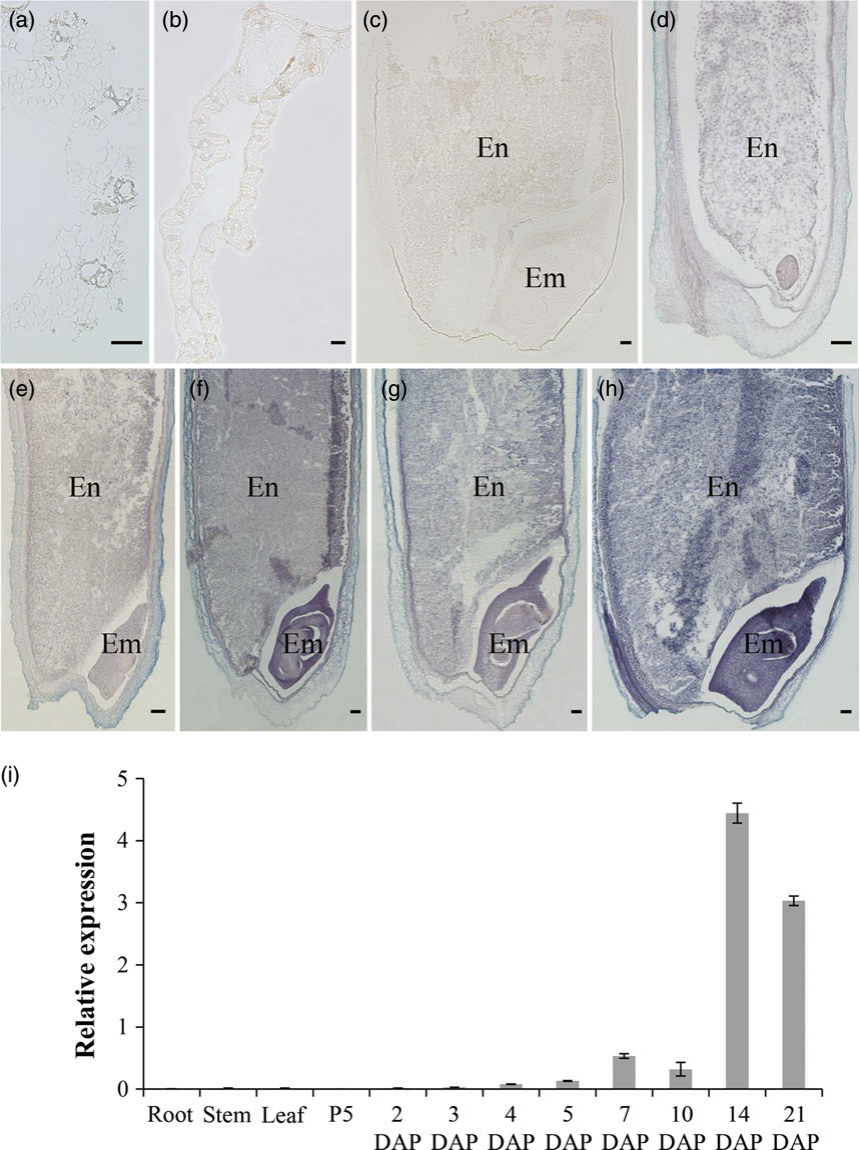
Spatial and temporal expression pattern of *RAG2*. (a–h) RNA *in situ* hybridization of *RAG2*. (a) Stem. (b) Leaf. (c) Sense. (d) 3‐d seed. (e) 5‐d seed. (f) 7‐d seed. (g) 10‐d seed. (h) 14‐d seed. En, Endosperm; Em, Embryo. Bar: 100 μm (a–h). (i) qRT‐PCR analysis of *RAG2*. Total RNAs were extracted from root (booting stages), stem (booting stages), leaf (1 day before heading), P5 is panicle at stage 5 (pollen mother cell formation stage), developing seeds (2, 3, 4, 5, 7, 10, 14 and 21 DAP). Data are mean ± SE for three replicates.

The expression pattern of *RAG2* was investigated in different tissues, including root, stem, leaf, panicle and seed of Zhonghua 11 (*Oryza sativa* ssp. *japonica* cv. Zhonghua 11). The analysis of qRT‐PCR indicated that *RAG2* was specifically expressed in the developing seed with a higher expression level during 14–21 DAP; however, the expression levels in leaf, root and stem were much lower (Figure 2i). These results suggest that *RAG2* may function in the developing seed of rice.

In addition, the temporal and spatial expression pattern of *RAG2* was determined by *in situ* hybridization (Figure 2a–h). The strong expression signal of *RAG2* was observed at 3, 5, 7, 10 and 14 DAP in seed, and gradually increased with seed development, which was consistent with the qRT‐PCR results (Figure 2d–h). In the 7‐, 10‐ and 14‐DAP endosperm, the expression signal was observed in aleurone layer (Figure 2f–h). In the 14‐DAP seed, the embryo and endosperm had the strongest hybridization signal of *RAG2* (Figure 2h). Above all, the expression of *RAG2* was specifically high in the developing seed, and it was strongly expressed in the developing embryo and endosperm. The temporal and spatial expression pattern of *RAG2* further suggested that it might play a role in rice seed.

### Production of *RAG2*‐overexpressed and *RAG2*‐suppressed lines

**Figure 3:**
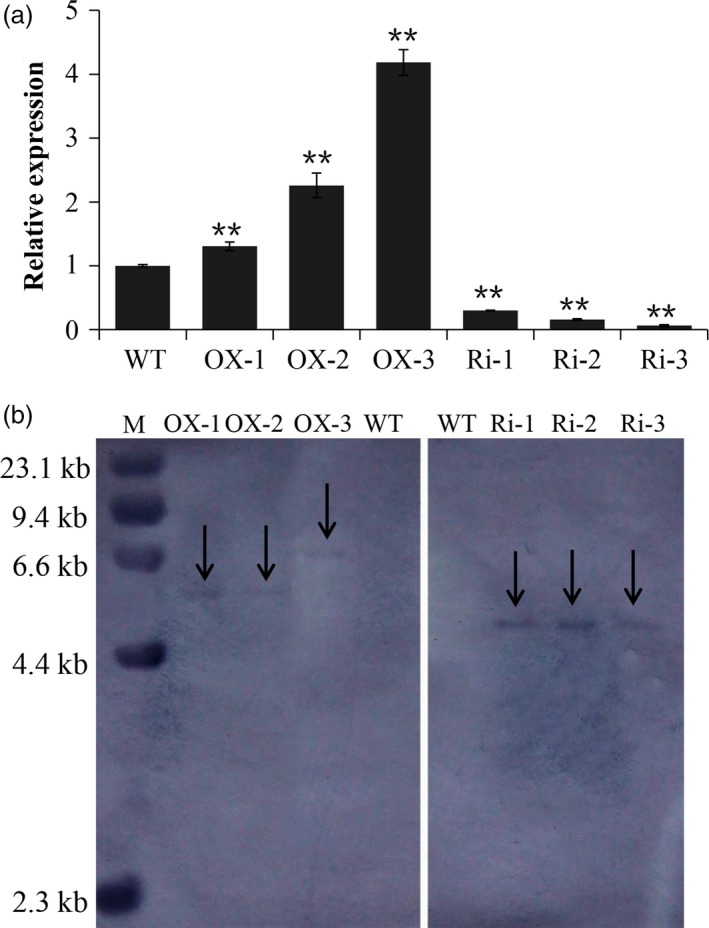
Analysis of WT and the transgenic lines. Real‐time quantitative RT‐PCR was used for analysis of *RAG2* transcription in transgenic rice (a), and 10 μg of genomic DNA from each plant line was digested with HindIII for DNA blot hybridization (b). Data are mean ± SE for three replicates. *P <* *0.05, **P <* *0.01. *P*‐values produced by two‐tailed Student's *t*‐test.

To investigate the function of *RAG2* in rice seed development, we generated transgenic rice with *RAG2* overexpression or suppression in the seed under the control of maize ubiquitin1 promoter. Successful transformants were confirmed by PCR. Twenty *RAG2*‐overexpressed lines (*RAG2*‐OX) and 45 *RAG2*‐suppressed lines (*RAG2*‐RNAi) were generated, respectively. Six lines (OX‐2, OX‐5, OX‐7, OX‐9, OX‐11 and OX‐15) with increased expression levels and six lines (Ri‐2, Ri‐7, Ri‐10, Ri‐17, Ri‐22 and Ri‐37) with reduced expression levels compared to WT were selected for the next generation. Additionally, three independent *RAG2*‐OX transgenic lines (OX‐2‐15, OX‐5‐5 and OX‐9‐22) with higher expression level, which originated from T_0_ generations (OX‐2, OX‐5 and OX‐9), were advanced to the T_2_ generation (Table S1) for functional analysis. Finally, three independent *RAG2*‐RNAi transgenic lines (Ri‐2‐21, Ri‐22‐12 and Ri‐37‐9) with lower expression level, which originated from T_0_ generations (Ri‐2, Ri‐22 and Ri‐37), were advanced to the T_5_ generation (Table S2) for functional analysis. To facilitate simplified drawing, three *RAG2*‐OX lines (OX‐2‐15, OX‐5‐5 and OX‐9‐22) with a higher expression level were renamed as OX‐1, OX‐2 and OX‐3, and three *RAG2*‐RNAi (Ri) lines (Ri‐22‐12, Ri‐2‐21 and Ri‐37‐9) with a lower expression level were renamed as Ri‐1, Ri‐2 and Ri‐3 (Figure 3a). DNA hybridization showed that pU2301‐Cflag (*RAG2*‐OX) and pDS1301‐*RAG2* (*RAG2*‐RNAi) were integrated into the genomes of transgenic plants as only one transgene copy, whereas no cross‐hybridization was observed in WT (Figure 3b).

Additionally, *RAG1* is the paralog of *RAG2* (Adachi *et al*., 1993), qRT‐PCR analysis showed that the expression level of *RAG1* was not affected in the RAG2‐RNAi transgenic lines (Figure S1).

### *RAG2* regulates seed size and grain yield

**Figure 4:**
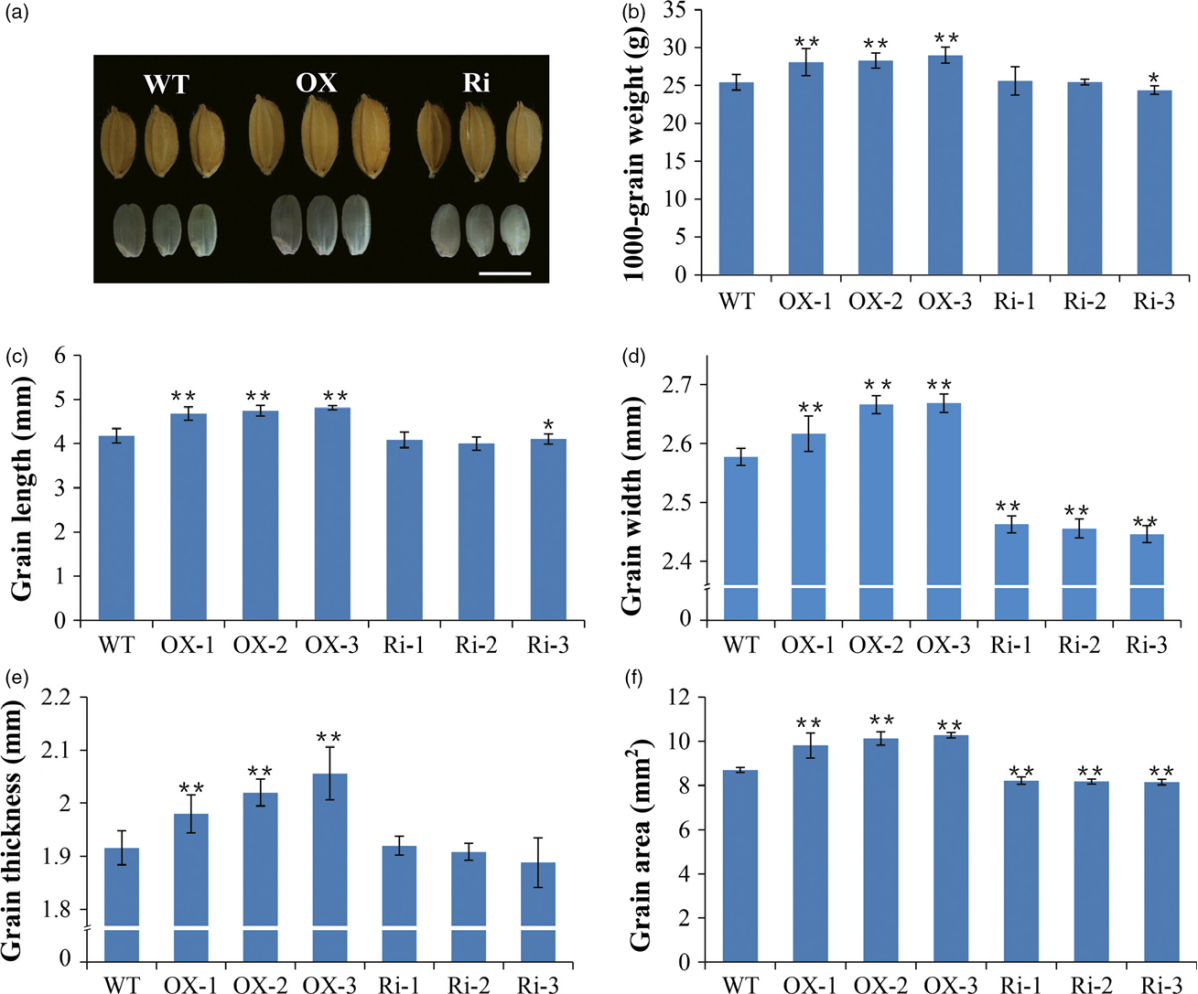
Phenotypes of grain yields of WT and the transgenic lines. (a) Grain size comparison among the *RAG2*‐OX,*RAG2*‐RNAi and WT seeds. (b) 1000‐grain weights of the *RAG2*‐OX,*RAG2*‐RNAi and WT seeds. (c) Grain lengths of the *RAG2*‐OX,*RAG2*‐RNAi and WT seeds. (d) Grain widths of the *RAG2*‐OX,*RAG2*‐RNAi and WT seeds. (e) Grain thicknesses of *RAG2*‐OX,*RAG2*‐RNAi and WT seeds. (f) Grain areas of the *RAG2*‐OX,*RAG2*‐RNAi and WT seeds. Scale bars (a) 5 cm. Values in (b–f) are means ± SD (n = 20). Two‐tailed Student's *t*‐tests were performed between the *RAG2*‐OX and WT, and *RAG2*‐RNAi and WT, respectively (*P <* *0.05, **P <* *0.01).

To investigate the influence of altered expression of the *RAG2* gene on yield‐related traits, we compared the seed setting rate and the 1000‐grain weight among WT, *RAG2*‐overexpressed (OX‐1, OX‐2 and OX‐3) and *RAG2*‐suppressed lines (Ri‐1, Ri‐2 and Ri‐3). Morphological/phenotypic differences were not observed between WT and the two types of transgenic lines at the vegetative stage. The *RAG2*‐OX lines and WT lines had the same seed setting rate and grain number per panicle. However, significant reductions were observed in the seed setting rate and grain number in the *RAG2*‐RNAi lines compared with the WT lines (Table 1). Notably, plants with overexpression of *RAG2* produced larger seeds than the RNAi and WT plants (Figure 4a, Figure S2). We found that *RAG2*‐OX transgenic plants showed a substantial increase in grain length compared to the WT (+12.0%, +13.6%, +15.2%, Figure 4c), with smaller increases in grain width (+1.5%, +3.4%, +3.5%, Figure 4d) and thickness (+3.3%, +5.4%, +7.3%, Figure 4e). We also detected a significant increase in seed area (+12.6%, +16.3%, +18.0%, Figure 4f) and 1000‐grain weight (+10.4%, +11.2%, +14.0%, Figure 4b) in *RAG2*‐OX transgenic plants. Meanwhile, *RAG2*‐RNAi transgenic plants exhibited an almost opposite phenotype compared to the WT, including a slight reduction in grain length (−2.2%, −1.9%, −1.8%, Figure 4c), grain thickness (+1.7%, −0.4%, −1.5%, Figure 4e) and 1000‐grain weight (+0.7%, −4.1%, +0.1%, Figure 4b); however, the decreases in seed area (−5.7%, −6.1%, −6.4%, Figure 4f) and grain width (−4.4%, −4.7%, −5.1%, Figure 4d) were clear. Furthermore, qRT‐PCR analysis revealed that a consistency existed between the expression level of *RAG2* and the phenotype of the seed, suggesting that regulation of the *RAG2* resulted in pleiotropic traits (Figures 3a and 4). Increased *RAG2* expression may possibly enlarge grain size by expanding the length, width and depth, hence, increasing grain weight.

**Table 1:** Analysis of yield parameters of WT and the transgenic lines

Line	No. of floret per panicles	No. of tillers per plant	Seed set rate (%)	1000‐grain weight (g)	Chalkiness rate (%)	Chalkiness degree
WT	156 ± 9	11.33 ± 1.18	82.38 ± 4.11	25.43 ± 1.04	10.49 ± 1.96	2.95 ± 0.08
OX‐1	150 ± 15	11.25 ± 1.16	79.09 ± 2.52	28.08 ± 0.78**	9.31 ± 3.18	3.84 ± 0.01**
OX‐2	151 ± 21	11.42 ± 0.95	84.71 ± 1.29	28.29 ± 1.03**	12.82 ± 0.02*	1.92 ± 0.03*
OX‐3	161 ± 18	11.33 ± 1.11	82.20 ± 5.90	29.00 ± 1.06**	6.03 ± 0.28*	2.66 ± 0.16
Ri‐1	147 ± 11	11.33 ± 1.03	70.13 ± 3.83**	25.617 ± 3.85	63.32 ± 4.14**	28.60 ± 0.28**
Ri‐2	141 ± 19*	11.17 ± 1.14	63.39 ± 7.17**	25.451 ± 0.54	70.69 ± 0.05**	31.14 ± 0.17**
Ri‐3	135 ± 16*	11.41 ± 1.04	64.09 ± 12.15**	24.391 ± 0.38*	73.99 ± 4.56**	32.64 ± 1.08**

Grains of the fully filled seeds were removed and used for analysis of the 1000‐grain weight. At least 400 grains of each plant were used to measure the grain chalkiness rate. Data are presented as means ± SE of three biological replicates.

*P <* *0.05, and **P <* *0.01 by two‐tailed Student's *t*‐test.

**Figure 5:**
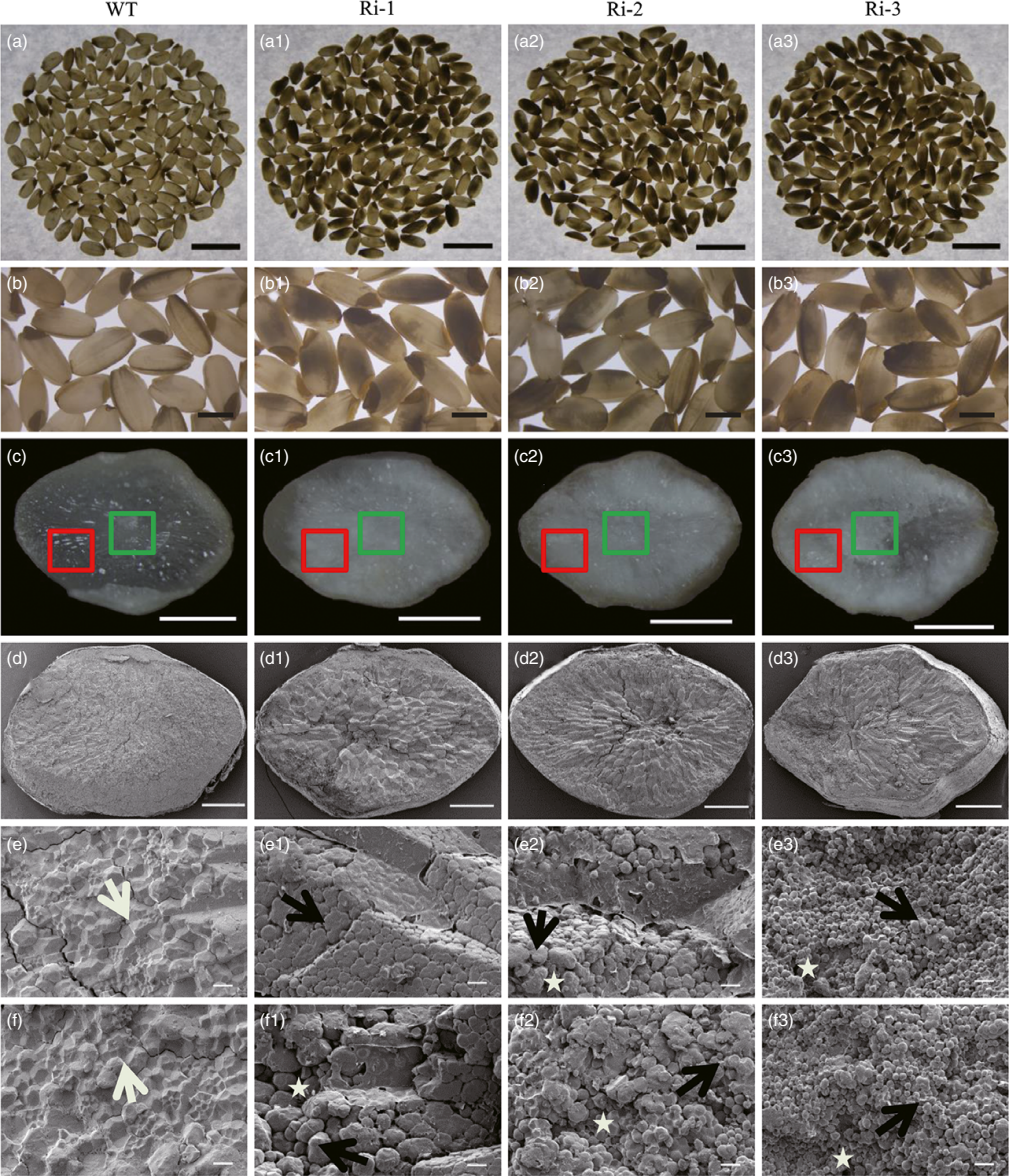
Phenotypes of seeds of WT and *RAG2*‐RNAi plant. WT: a, b, c, d, e, f. Ri‐1: a1, b1, c1, d1, e1, f1. Ri‐2: a2, b2, c2, d2, e2, f2. Ri‐3: a3, b3, c3, d3, e3, f3. (a‐a3) 150 grains of mature seeds in white light background. (b‐b3) Mature seeds. (c–c3) Cross sections of mature endosperm. *RAG2*‐RNAi line grains displayed higher chalkiness with less translucence compared with the WT. (d–d3) SEM of the central area of mature endosperm in (c–c3). (e–e3) SEM of the central area of mature endosperm, with the cross sections indicated by a green square in (c–c3). (f–f3) SEM of the central area of mature endosperm, with the cross sections indicated by a red square in (c–c3). White and black arrows in (e–e3, f‐f3) indicate polygonal and round starch granules, respectively. Areas in which starch granules are loosely packed are indicated by asterisks. The packaging of starch granules in the endosperm of *RAG2*‐RNAi lines (Ri‐1, Ri‐2 and Ri‐3) was loose, with round and smaller granules. Scale bars: 10 mm (a–a3), 3 mm (b–b3), 1 mm (c–c3), 500 μm (d–d3), 10 μm (e–e3, f–f3). Data are mean ± SE for three replicates. *P <* *0.05, **P <* *0.01. *P*‐values produced by two‐tailed Student's *t*‐test.

We also observed that the grains in *RAG2*‐RNAi lines displayed higher chalkiness compared with the WT (Table 1). The results of scanning electron microscopy (SEM) indicated that the starch granules in *RAG2*‐RNAi lines were loosely packed and irregularly polyhedron‐shaped compared with those in the WT (Figure 5), while in *RAG2*‐OX lines, starch granules had irregular polyhedron shapes similar to those of WT granules (Table 1, Figure S3, e1–e3, f1–f3). These results suggest that *RAG2* might also be involved in chalky endosperm formation.

### *RAG2* affects the accumulation of seed storage substances

To analyse the significant increase in grain weight in *RAG2*‐OX lines and the effect of *RAG2* on grain quality, we measured the contents of total protein, starch and total lipid.

**Figure 6:**
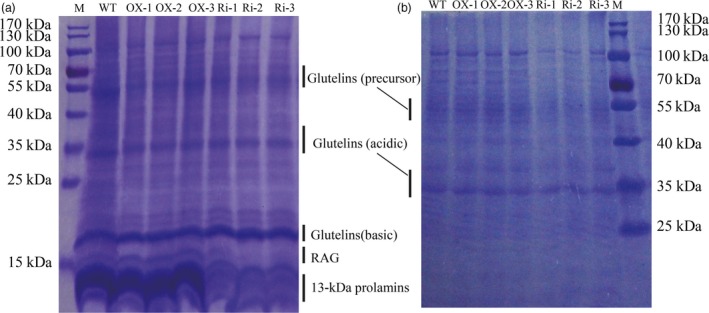
Seed storage proteins level in seeds of WT and the transgenic lines. SDS‐PAGE of seed proteins extracted from mature seeds. Glutelins (precursor, acidic and basic subunits), α‐globulin, 14‐to‐16‐kDa rice protein (RAG) and 13‐kDa prolamins are indicated. a: Add 10 μL sample; b: Add 5 μL sample.

**Figure 7:**
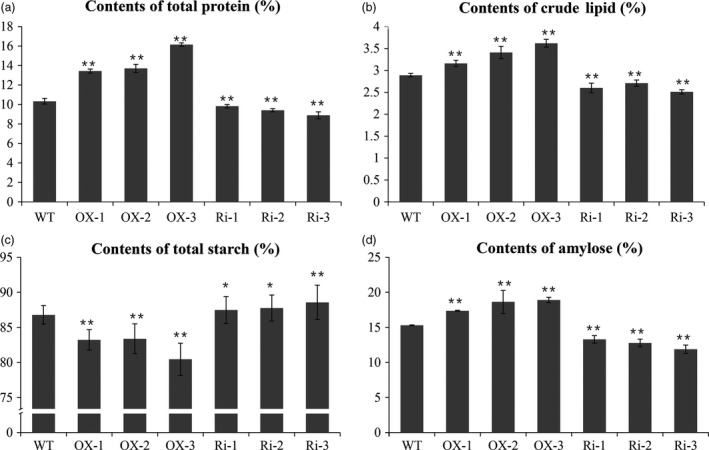
Comparisons of protein, lipid and starch contents in mature seeds of WT and the transgenic lines. Powder grains of fully filled seeds of the WT and the transgenic lines were used for analysis of the starch, protein and lipid content. (a) Contents of total protein. (b) Contents of total starch. (c) Contents of crude lipid. (d) Contents of amylose. Data are mean ± SE for three replicates. *P <* *0.05, **P <* *0.01. *P*‐values produced by two‐tailed Student's *t*‐test.

Measurement by the chemical method revealed that the content of seed total protein was increased by 3, 4.8 and 4 mg/g in the OX‐1, OX‐2 and OX‐3 lines and reduced by 1.9, 2.4 and 1.6 mg/g in the Ri‐1, Ri‐2 and Ri‐3 lines, respectively, compared with the WT (Figure 7a, Table 2). The glutelin and prolamin contents were clearly increased in the *RAG2*‐OX lines (+28.5%, +31.0%, +55.5%) and reduced in the *RAG2*‐RNAi lines (+1.6%, −2.5%, −7.9%) (Table 2). Based on the SDS‐PAGE analysis, most of the SSPs, such as the glutelins and prolamins, were higher in the *RAG2*‐OX lines and lower in the *RAG2*‐RNAi lines (Figure 6). Previous research showed that RAG2 was an albumin. The 16‐kDa RAG2 was increased in the *RAG2*‐OX lines and almost disappeared in the *RAG2*‐RNAi lines (Figure 6a).

**Table 2:** Contents of protein, starch and lipid in mature seeds

Line	Contents of total protein (mg/g)	Contents of prolamin (%)	Contents of glutelin (%)	Contents of total starch (%)	Contents of total lipid (%)
WT	102.14 ± 0.79	0.33 ± 0	10.00 ± 0.29	86.79 ± 1.33	2.89 ± 0.04
OX‐1	105.16 ± 0.65**	0.57 ± 0.01**	12.85 ± 0.20**	83.22 ± 1.46**	3.16 ± 0.07**
OX‐2	106.15 ± 1.14**	0.59 ± 0.02**	13.10 ± 0.42**	83.37 ± 2.13**	3.41 ± 0.14**
OX‐3	106.94 ± 0.87**	0.60 ± 0.02**	15.55 ± 0.16**	80.45 ± 2.30**	3.62 ± 0.09**
Ri‐1	100.55 ± 0.37**	0.30 ± 0.03**	10.16 ± 0.20	87.47 ± 1.92*	2.60 ± 0.11**
Ri‐2	99.79 ± 0.46**	0.32 ± 0.02*	9.75 ± 0.18*	87.76 ± 1.86*	2.71 ± 0.07*
Ri‐3	100.22 ± 0.31**	0.31 ± 0.02*	9.21 ± 0.38**	88.57 ± 2.45**	2.51 ± 0.05**

Powder grains of the transgenic lines and WT fully filled seeds were used for analysis of the lipid, protein and starch contents. All data are given as means ± SE of three biological replicates. *P <* *0.05. **P <* *0.01. *P*‐values produced by two‐tailed Student's *t*‐test.

Total starch content and apparent amylose content (AAC) were measured in transgenic rice seeds. Total starch content in Ri‐1 (88.57%), Ri‐2 (87.76%), Ri‐2 (87.47%), OX‐1 (83.22%), OX‐2 (83.37%) and OX‐3 (80.45%) was largely changed compared to the WT (86.79%; Figure 7c, Table 2). The AAC was lower in Ri‐1 (11.88%), Ri‐2 (12.78%) and Ri‐2 (13.29%), but higher in OX‐1 (17.37%), OX‐2 (18.63%) and OX‐3 (18.91%) compared with the WT (15.30%) (Figure 7d, Table 2). Thus, the AAC was higher, by 12.8%–24.3%, in the *RAG2*‐OX lines and lower, by 19.6%–26.4%, in the *RAG2*‐RNAi lines than in the WT.

The results demonstrate that the total lipid content of the seed in three *RAG2*‐OX lines (OX‐1, OX‐2 and OX‐3) was 27.70%, 28.33% and 31.52% higher than that in the WT. Conversely, the total lipid content of the seed was 10.03%, 6.22% and 13.15% lower in the three *RAG2*‐RNAi lines (Ri‐1, Ri‐2 and Ri‐3) (Figure 7b, Table 2). Crude FA components of rice include palmitic, oleic and linoleic acids, among others. The palmitic acid is a saturated FA with the highest content in rice seed (Ying *et al*., 2012), and the oleic acid is a predominant monounsaturated FA of rice seed oil. In view of this, we further tested the content of six main FAs composition using GC‐MS. The results showed that C16:0 (palmitic acid), C18:1 (oleic acid), C18:2 (linoleic acid) and C18:3 (linolenic acid) were significantly increased in the three *RAG2*‐OX lines, but decreased in the three *RAG2*‐RNAi lines (Table 3).

**Table 3:** Contents of fatty acid in mature seeds

Traits	Fatty acid composition (mg/g)						
	WT	OX‐1	OX‐2	OX‐3	Ri‐1	Ri‐2	Ri‐3
C16:0 (Palmitic acid)	4.84 ± 0.06	5.12 ± 0.05*	5.38 ± 0.08**	5.90 ± 0.02**	4.53 ± 0.02**	4.25 ± 0.01**	4.04 ± 0.01**
C18:0 (Stearic acid)	0.59 ± 0.03	0.60 ± 0.02	0.67 ± 0.06**	0.76 ± 0.04**	0.64 ± 0.03	0.64 ± 0.01	0.58 ± 0.03
C18:1 (Oleic acid)	12.52 ± 0.21	13.52 ± 0.16**	14.36 ± 0.38**	14.87 ± 0.15**	10.34 ± 0.69**	9.98 ± 0.31**	9.85 ± 0.20**
C18:2 (Linoleic acid)	10.23 ± 0.13	11.57 ± 0.08**	12.84 ± 0.39**	13.72 ± 0.14**	10.33 ± 0.40	10.42 ± 0.26	10.01 ± 0.05*
C18:3 (Linolenic acid)	0.36 ± 0.02	0.42 ± 0.02**	0.46 ± 0. 01**	0.48 ± 0.02**	0.38 ± 0.01	0.37 ± 0.02	0.35 ± 0.01
C20:0 (Eicosanoic acid)	0.22 ± 0.01	0.24 ± 0.01	0.45 ± 0.07**	0.27 ± 0.02**	0.20 ± 0.03*	0.20 ± 0.04*	0.18 ± 0.02**

Data are presented as means ± SE of three biological replicates.

*P <* *0.05. **P <* *0.01. *P*‐values produced by two‐tailed Student's *t*‐test.

### Relative expression levels of the genes related to seed storage compounds

Storage compounds were altered in *RAG2*‐overexpressed and *RAG2*‐suppressed seeds, and we investigated the expression level of the genes related to these compounds. Total RNA for qRT‐PCR was extracted from the developing seeds at 14 DAF.

**Figure 8:**
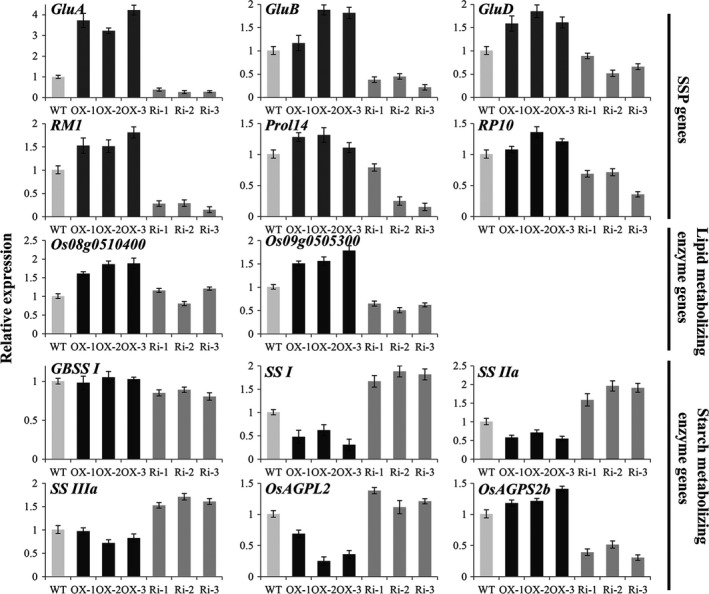
Expression analyses of SSP genes and starch and lipid‐metabolizing enzyme genes by quantitative RT‐PCR. RNA was extracted from developing seeds at 14 d after flowering (DAF) for SSP genes (*GluA*,* GluB*,* GluD*,*RM1*,* Prol14* and *RP10*) and lipid (*Os08g0510400* and *Os09g0505300*) and starch (*GBSS I*,*SS I*,*SS IIa*,*SS IIIa*,* OsAGPL2* and *OsAGPS2b*)‐metabolizing enzyme genes. Light‐grey, black and dark‐grey bars represent the expression levels in WT,*RAG2*‐OX and *RAG2*‐RNAi line seeds, respectively. The relative expression levels were normalized to those of *UBI*.

The expression levels of SSP genes were closely correlated with their protein level in each line (Figures 6 and 8). In the *RAG2*‐OX lines, the glutelin gene *GluA* was obviously increased, and the *GluB* and *GluD* were slightly enhanced (Figure 8). Conversely, in the *RAG2*‐RNAi lines, the levels of *GluA* and *GluB* were notably reduced, and those of *GluD* were slightly decreased compared to the WT (Figure 8). The prolamin genes, such as *RM1* (Cys‐rich 13‐kDa prolamin), *Prol14* (Cys‐poor 13‐kDa prolamin) and *RP10* (10‐kDa prolamin), were slightly increased in the *RAG2*‐OX lines and decreased in the *RAG2*‐RNAi lines. These results further suggest that the changes in expression of *RAG2* led to altered expression of rice SSP genes.

We examined the expression level of starch‐related genes. The expression levels of the granule‐bound starch synthase gene *GBSS I* and the ADP glucose pyrophosphorylase gene *OsAGPS2b* were decreased in the *RAG2*‐RNAi line seeds and were comparable in the *RAG2*‐OX lines and the WT (Figure 8). The expression of genes encoding other starch‐metabolizing enzymes was increased in seeds in the *RAG2*‐RNAi lines and decreased in the *RAG2*‐OX lines. These findings are consistent with measurement of the previous components, suggesting that *RAG2* influences starch biosynthesis.

We also examined the expression levels of the rice ketoacyI‐ACP reductase gene *Os08g0510400* and the acyIACP thioesterase gene *Os09g0505300*, which are known to play crucial roles in lipid metabolism in *Arabidopsis*, and are rice homologues of *Arabidopsis At1g62610* and *At3g25110*, respectively (Mu *et al*., 2008). The expression of *Os08g0510400* and *Os09g0505300* was markedly increased in *RAG2*‐OX line seeds and decreased slightly in *RAG2*‐RNAi line seeds (Figure 8). These results suggest that *RAG2* influences the accumulation of fat.

A change in *RAG2* expression leads to the altered expression of storage substance genes. These results are also consistent with the change in storage components.

## Discussion

In the study, we demonstrated that *RAG2*, a 16‐kDa α‐amylase/trypsin inhibitor of rice, was expressed specifically in the developing seed and was possibly involved in the regulation of grain yield and grain quality of rice. The overexpression of *RAG2* in transgenic rice was found to increase the content of storage proteins and lipids in seeds and to improve grain yield. Thus, our results may help future rice molecular breeding focused on improving grain yields and seed quality.

In this study, overexpression of *RAG2* on the background of WT remarkably increased grain size (Figure 4, Figure S2) and 1000‐grain weight (Figure 4b, Table 1), while RNAi‐mediated knockdown of *RAG2* resulted in decreased grain size (Figure 4, Table 1). *GS3*,* GS5*,* GL3*,* GW2* and *GW8* have been reported to control the grain weight by regulating grain size in rice (Fan *et al*., 2006; Li *et al*., 2011; Song *et al*., 2007; Tan *et al*., 2000; Wang *et al*., 2012; Zhang *et al*., 2012). However, the expression level of these genes was not significantly different between the WT and transgenic plants (Figure S4). Furthermore, histological analysis showed no obvious changes in the morphology phenotype of endosperm cells among WT, *RAG2*‐OX and *RAG2*‐RNAi lines (Figure S5). These results suggest that *RAG2* regulates rice grain weight in a pathway distinct from those associated with previous cloned grain size regulators in rice. We speculated that *RAG2* regulated grain weight by influencing the degree of grain filling (plumpness).

Grain plumpness is determined mainly by the accumulation of storage substances, such as starch, proteins and lipids (Zuo and Li, 2014). Previous studies showed that change in storage substance accumulation affected the grain size and grain weight. *FLO2* as a transcription factor actives starch synthesis‐related genes for grain size and grain weight regulation in rice. Overexpression of *FLO2* enlarged the size and weight of grains significantly (She *et al*., 2010). *GIF1* encodes a cell‐wall invertase that is required for carbon partitioning during early grain filling. Overexpression of *GIF1* increases grain size and grain weight (Wang *et al*., 2008). Loss function of the *TGW6* allele increases grain weight through pleiotropic effects on source organs and results in significant yield increases (Ishimaru *et al*., 2013). Similarly, overexpression of the *RAG2* gene was found to increase the content of storage proteins and lipids in transgenic rice. Therefore, overexpression of *RAG2* may possibly improve the accumulation of seed storage substances and contribute to enlarging the size and weight of grains in rice. Further supports were from the expression pattern of *RAG2*. qRT‐PCR and *in situ* hybridization showed that *RAG2* was specifically high expressed in seed at 14–21 DAP. This stage is an important period of accumulation of seed storage substances.

Rice is the main source of people's daily intake of protein, providing them with a total amount of protein that is three times that of beans (Mahmoud and El Anany, 2014). The content and composition of SSPs are important nutrient qualities of seeds (Kim *et al*., 2013). Research by the International Rice Research Institute (IRRI) showed that the average content of SSPs was 9.5% in rice (Gomez, 1979). Here, we found that overexpression of *RAG2* increased total protein content by 3 mg/g to 4.8 mg/g. The content of glutelin and prolamin was significantly increased in the *RAG2*‐OX lines. The glutelin storage protein is an important digestible protein and is an index of nutrient quality of seeds (Kim *et al*., 2013). Moreover, qRT‐PCR analysis found that the expression level of most of the SSP‐related genes, including glutelin genes (*GluA*,* GluB* and *GluD*) and prolamin genes (*RM1*,* Prol14* and *RP10*), was increased in the *RAG2*‐OX lines and decreased in the *RAG2*‐RNAi lines. These results suggest that *RAG2* may involve in the accumulation of SSP in rice. Overexpression of *RAG2* increased the protein content, thereby increasing the nutritional quality of seeds.

The FA content is another nutritional quality of seeds that determines the appearance and eating quality of rice (Boxer *et al*., 2010). The content of total lipids was increased (9%–25%) in the *RAG2*‐OX line seeds, but decreased in the *RAG2*‐RNAi line seeds (Figure 7a–c, Table 2). A recent report showed that OsLTPL36, a lipid‐transfer protein of rice, is essential for FA accumulation and seed quality (Wang *et al*., 2015). The analysis of the homologous sequence showed that the 19 amino acid residues of lipid‐transfer proteins are like those present in RAG2 of rice (Adachi *et al*., 1993), implying that *RAG2* might be important in lipid metabolism for FA accumulation in rice seed.

The main seed storage substances of rice include starch, proteins and lipids (Fitzgerald *et al*., 2009). The total percentage of seed storage substances is constant, and a change in one component will certainly lead to a change in the percentage of the other contents (Fitzgerald *et al*., 2009; Zhu *et al*., 2003). *OsPPDKB* encodes a pyruvate orthophosphate dikinase that modulates carbon metabolism during grain filling in rice. The starch content is reduced, the total protein content is slightly higher, and the lipid is significantly increased in its mutant (*flo4*) (Kang *et al*., 2005). In a sweet wheat (*SW*) mutant lacking functional granule‐bound starch synthase I (GBSSI) and starch synthase IIa (SSIIa), the starch content is much lower, but the content of protein and lipid is higher compared to the WT (Shimbata *et al*., 2011). Similarly, our results showed that a change in the expression of *RAG2* not only affected the accumulation of storage proteins, but also affected the lipid and starch contents.

A previous study showed that several rice proteins, such as 14‐to‐16‐, 26‐, 33‐ and 60‐kDa proteins, were recognized by serum IgE of patients showing hypersensitive reactions to rice ingestion (Tsukasa Matsuda *et al*., 1991). As the first reported allergen protein in rice (Ito *et al*., 2005), the protein content of RAG2 was obviously decreased in the *RAG2*‐RNAi lines (Figure 6), but the main nutritional protein glutelin was decreased less. In conclusion, although the content of the allergen protein RAG2 was increased in *RAG2*‐OX lines, both the grain yield and the nutritional quality were improved. This kind of transgenic rice, with high nutritional value and high yield, could be provided to the vast majority of nonallergic people. While in the *RAG2*‐RNAi lines, the content of RAG2 protein was obviously decreased, this type of transgenic rice, with lower allergen protein, could be a functional food to allergic patients.

## Experimental procedures

### Plasmid construction and transformation of rice

To construct the overexpression vector of *RAG2*, a 498‐bp cDNA fragment (accession number AK107328) without its stop codon encoding the full length of *RAG2* was amplified, and was then linked to pU2301‐cFLAG, which carries a maize ubiquitin promoter.

To construct the RNA interference vector of *RAG2*, a 394‐bp cDNA fragment of *RAG2* was amplified, and was then inserted into pDS1301. The recombinant with the first intron, splicing acceptor site and nos terminator gene was driven by maize ubiquitin promoter.

The recombinant constructs were introduced into *Agrobacterium tumefaciens* strain EHA105 that were further transferred into Zhonghua 11 (ZH11) (*Oryza sativa* ssp. *japonica*) as reported (Ma *et al*., 2015). Primers used in this study are shown in Table S3.

### Plant materials and evaluation of the yield‐related traits

*RAG2*‐FLAG overexpression transgenic plants and *RAG2*‐RNAi transgenic plants were generated in Zhonghua11 (*Oryza sativa* ssp. *japonica* cv. Zhonghua11) background. All the requisite rice plants were grown in a field at the Huazhong Agricultural University, Wuhan and Hainan, China.

Fully filled grains were used for measuring grain size (length, width and thickness), grain quality and 1000‐grain weight. Grain size (length, width and area) of nearly 400 grains from each line was measured using a MRS‐9600TFU2L (MICROTEK) grain observation instrument. Twenty randomly chosen grains from each plant were lined up length‐wise and width‐wise along a Vernier caliper to measure grain length and grain width, respectively. Grain thickness of 20 randomly chosen grains from each plant was measured using Vernier caliper. Grain chalkiness was assessed by the MRS‐9600TFU2L (MICROTEK) grain observation instrument. Grain weight was calculated on the basis of 200 grains and then converted to 1000‐grain weight as previously described (Li *et al*., 2014). All of the experimental grains were dehulled grains, except the 1000‐grain weight grains. Phenotypic measurements of the positive transgenic plants were undertaken using three independent lines at least.

### Sequence and structural analysis of *RAG2*

DNA and protein sequence of *RAG2* were obtained from MSU database (http://rice.plantbiology.msu.edu/) and KOME database (http://cdna01.dna.affrc.go.jp/cDNA/). Blast similarity protein sequence according to the following criteria: query cover>30%, identity>40%, E value<1e‐5 in NCBI (http://www.ncbi.nlm.nih.gov/). Proteins are shown in the figure with the corresponding accession numbers. Alignments were performed in ClustalW.

### RNA isolation and quantitative RT‐PCR analyses

Total RNA of various samples was extracted using the TRIzol reagent (Invitrogen, Waltham, MA). First‐strand cDNA was synthesized using PRIME Script Reverse Transcriptase (TaKaRa, Dalian, China). The expression levels were measured using an ABI StepOne™ Real‐time System (Applied Biosystems, Carlsbad, CA) with rice *Ubiquitin* as the internal control. Primers used in this study are shown in Table S3.

### *In situ* hybridization

Paraffin sections were obtained according to the previous method (Ma *et al*., 2015). A 135‐bp specific fragment was obtained and cloned into pGM‐T vector. Hybridization of digoxigenin‐labelled antisense and sense probes and immunological detection were performed as described (Sang *et al*., 2012). The primers are listed in Table S3.

### DNA blot hybridization

Southern blotting was performed as previously described (Chen *et al*., 2015). DNA probes specific to G418 (for pU2301‐*RAG2*‐cFLAG) and hph (for pDS1301‐*RAG2*) coding sequence were labelled using a PCR DIG probe synthesis kit (Roche Diagnostics). Detection of the signals was carried out using a Nikon camera (E60, Japan). The primers are listed in Table S3.

### Scanning electron microscopy

The samples were transversely sectioned, natural dried, sputter‐coated with gold particles, observed and photographed using a scanning electron microscope (JSM‐6390LV; JEOL, Japan).

### SDS‐PAGE

SDS‐PAGE was carried out using total proteins from seeds as per previous method (Yamamoto *et al*., 2006). After electrophoresis, SDS‐PAGE gels were stained using Coomassie Brilliant Blue G‐250 (Nacalai Tesque, http://www.nacalai.co.jp).

### Statistical analysis

Data were subjected to a software package used for statistical analysis, and significant differences between individual means were established using a two‐tailed Student's *t*‐test in Microsoft Office Excel 2010. Significance was accepted at *P *<* *0.05 and *P *<* *0.01.

### Analysis of the total protein, starch and total lipid contents

Hundred milligram, 500 mg and 3.4 g of grounded rice powder were used for the assays of total protein, starch and total lipid contents, respectively. All measurements were performed in three biological replicates.

Total protein content was detected using an XDS Near‐Infrared Rapid Content Analyzer (Foss^®^ Analytical, Hilleroed, Denmark) (Li *et al*., 2014). The content of glutelin and prolamin was measured using the Bradford assay (Yang *et al*., 2013). Starch content was measured as previously described method (Li *et al*., 2014). Crude fat content was measured using the Soxtec method (Hijona *et al*., 2010). FA extraction was performed as previously described (Pan *et al*., 2011). Quantification of FA content was measured using GC‐MS (HP7890, California) (Ying *et al*., 2012).

## Conflict of Interest

The authors declare no conflict of interests.

## Supporting information


**Figure S1** Expression analyses of *RAG1* by quantitative RT‐PCR. RNA was extracted from developing seeds at 14 d after flowering (DAF). Light‐grey, black, and dark‐grey bars represent the expression levels in WT, *RAG2*‐OX, and *RAG2*‐RNAi line seeds, respectively. The relative expression levels were normalized to that of *UBI*.

**Figure S2** Comparison of mature seeds of WT and the transgenic lines. (a) Comparison of 20 mature seeds length and width of WT, *RAG2*‐OX, and *RAG2*‐RNAi lines. Bar = 1 cm. (b) Statistics of 20 mature seeds length and width of WT, *RAG2*‐OX, and *RAG2*‐RNAi lines. Data are mean ± SE for three replicates. *P <* *0.05, **P <* *0.01. *P*‐values produced by two‐tailed Student's *t*‐test.

**Figure S3** Phenotypes of seeds of WT and *RAG2*‐OX plant. WT: a, b, c, d, e, f. Ri‐1: a1, b1, c1, d1, e1, f1. Ri‐2: a2, b2, c2, d2, e2, f2. Ri‐3: a3, b3, c3, d3, e3, f3. (a‐a3) 150 grains of mature seeds in white light background. (b‐b3) Mature seeds. (c–c3) Cross sections of mature endosperm. *RAG2*‐OX lines grains displayed comparable chalkiness with WT. (d–d3) SEM of the central area of mature endosperm in (c–c3). (e–e3) SEM of the central area of mature endosperm, with the cross sections indicated by a green square in (c–c3). (f–f3) SEM of the central area of mature endosperm, with the cross sections indicated by a red square in (c–c3). Scale bars: 10 mm (a–a3), 3 mm (b–b3), 1 mm (c–c3), 500 μm (d–d3), 10 μm (e–e3, f–f3). (g) Grain chalkiness rate of WT and 3 RNAi lines. Data are mean ± SE for three replicates. *P <* *0.05, **P <* *0.01. *P*‐values produced by two‐tailed Student's *t*‐test.

**Figure S4** Expression analyses of grain weight and grain size genes by quantitative RT‐PCR. RNA was extracted from developing seeds at 14 d after flowering (DAF) for these genes (*GS3*,* GS5*,* GL3*,* GW2*, and *GW8*). Light‐grey, black, and dark‐grey bars represent the expression levels in WT, *RAG2*‐OX, and *RAG2*‐RNAi line seeds, respectively. The relative expression levels were normalized to that of *UBI*.

**Figure S5** Longitudinal section showing seed development of *RAG2*‐OX lines, *RAG2*‐RNAi lines and corresponding wild‐type plants. Longitudinal section seeds were stained with 0.05% haematoxylin to compare seed development in wild‐type Zhonghua11 (ZH11), *RAG2*‐OX and *RAG2*‐RNAi lines. Bars = 300 μm. Em, embryo; En, endosperm.

**Table S2** Analysis of yield parameters of WT and *RAG2*‐RNAi T_1_ lines.

**Table S3** Primers used for functional analysis of *RAG2*.

**Table S1** Analysis of yield parameters of WT and *RAG2*‐OX T_1_ lines.
