# Supplementary figures and images for: Overexpression of the 16‐kDa α‐amylase/trypsin inhibitor RAG2 improves grain yield and quality of rice
Source: Plant Biotechnol J. 2016 Nov 22;15(5):568–80. doi: 10.1111/pbi.12654 (PMC5399008; doi:10.1111/pbi.12654)

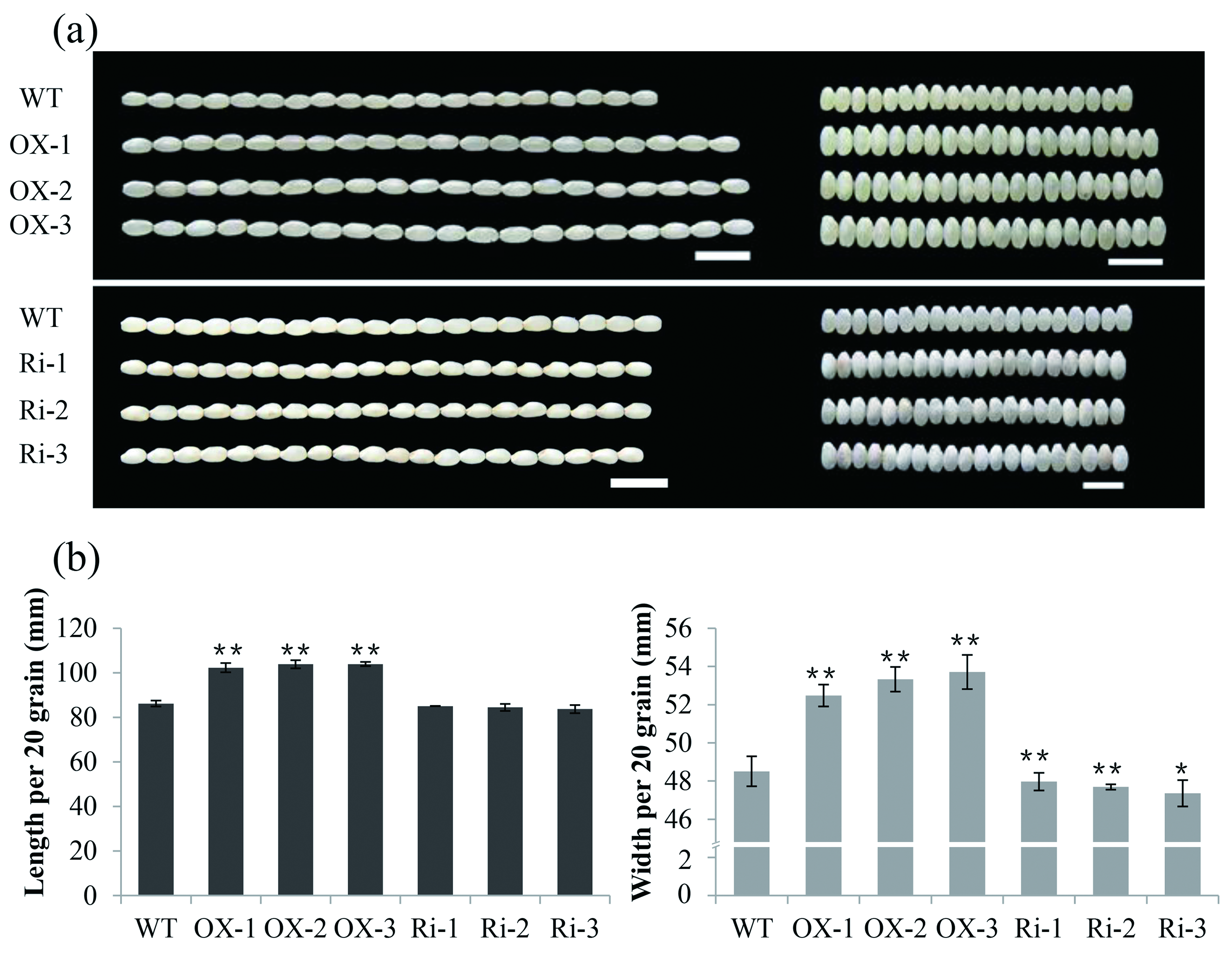

Supplement: Supplementary file 2 — Figure S2 Comparison of mature seeds of WT and the transgenic lines. (a) Comparison of 20 mature seeds length and width of WT, RAG2‐OX, and RAG2‐RNAi lines. Bar = 1 cm. (b) Statistics of 20 mature seeds length and width of WT, RAG2‐OX, and RAG2‐RNAi lines. Data are mean ± SE for three replicates. *P < 0.05, **P < 0.01. P‐values produced by two‐tailed Student's t‐test. [file PBI-15-568-s006.tif]

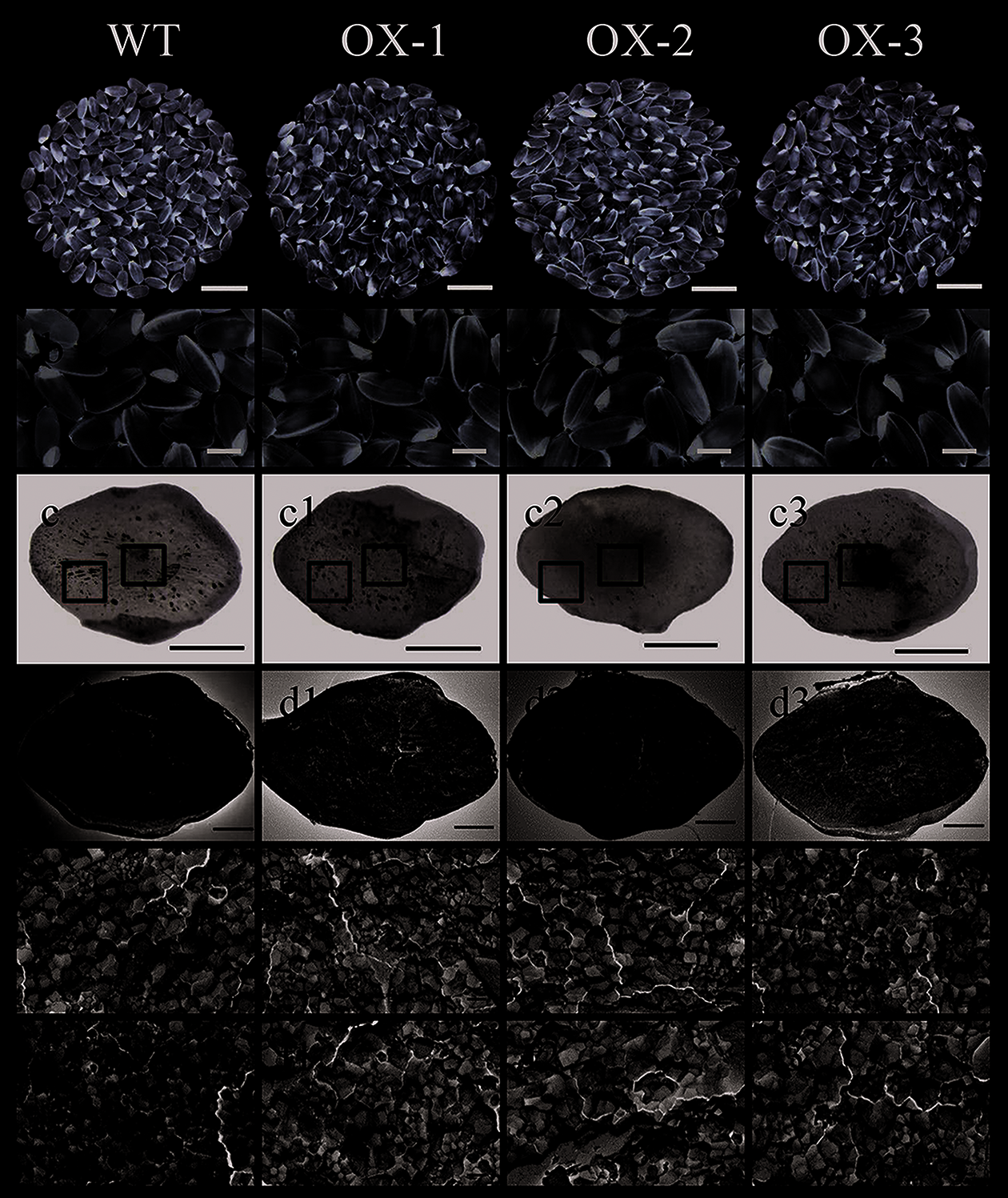

Supplement: Supplementary file 3 — Figure S3 Phenotypes of seeds of WT and RAG2‐OX plant. WT: a, b, c, d, e, f. Ri‐1: a1, b1, c1, d1, e1, f1. Ri‐2: a2, b2, c2, d2, e2, f2. Ri‐3: a3, b3, c3, d3, e3, f3. (a‐a3) 150 grains of mature seeds in white light background. (b‐b3) Mature seeds. (c–c3) Cross sections of mature endosperm. RAG2‐OX lines grains displayed comparable chalkiness with WT. (d–d3) SEM of the central area of mature endosperm in (c–c3). (e–e3) SEM of the central area of mature endosperm, with the cross sections indicated by a green square in (c–c3). (f–f3) SEM of the central area of mature endosperm, with the cross sections indicated by a red square in (c–c3). Scale bars: 10 mm (a–a3), 3 mm (b–b3), 1 mm (c–c3), 500 μm (d–d3), 10 μm (e–e3, f–f3). (g) Grain chalkiness rate of WT and 3 RNAi lines. Data are mean ± SE for three replicates. *P < 0.05, **P < 0.01. P‐values produced by two‐tailed Student's t‐test. [file PBI-15-568-s005.tif]

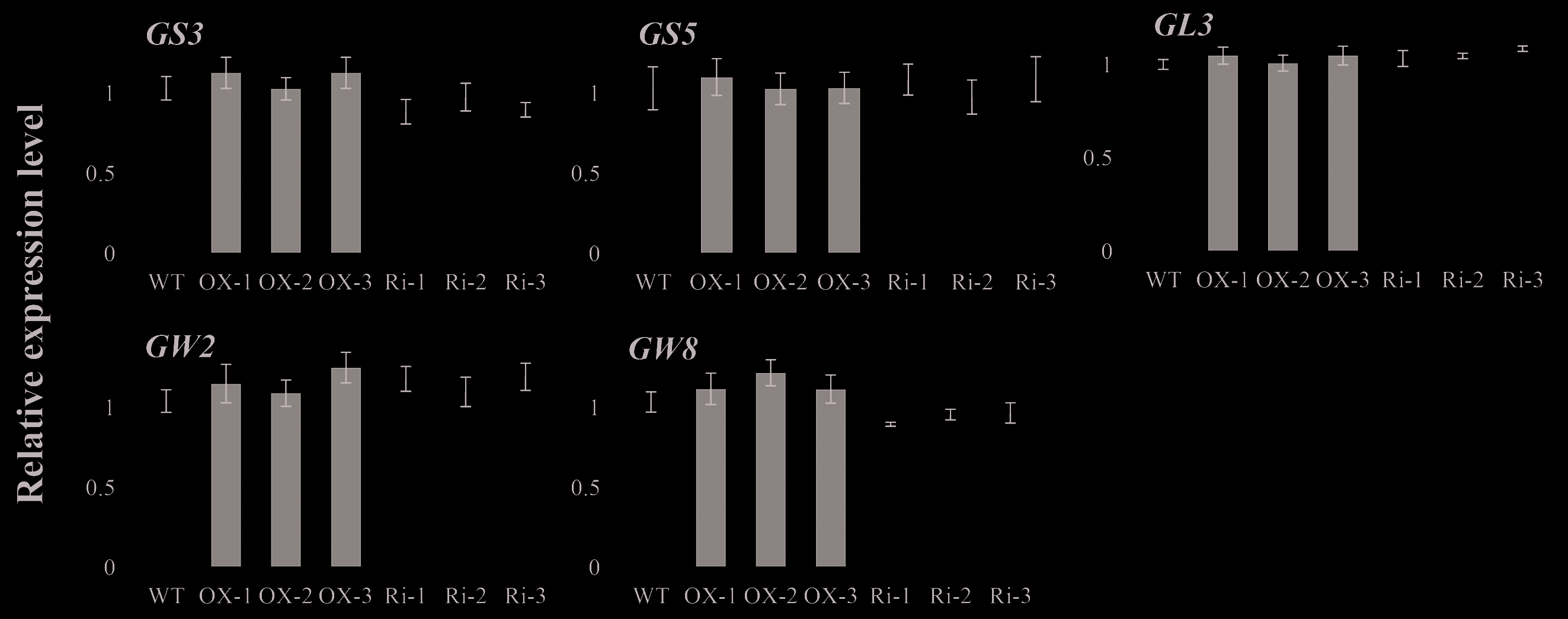

Supplement: Supplementary file 4 — Figure S4 Expression analyses of grain weight and grain size genes by quantitative RT‐PCR. RNA was extracted from developing seeds at 14 d after flowering (DAF) for these genes (GS3, GS5, GL3, GW2, and GW8). Light‐grey, black, and dark‐grey bars represent the expression levels in WT, RAG2‐OX, and RAG2‐RNAi line seeds, respectively. The relative expression levels were normalized to that of UBI. [file PBI-15-568-s007.tif]
