# Supplementary material for: Overexpression of the 16‐kDa α‐amylase/trypsin inhibitor RAG2 improves grain yield and quality of rice
Source: Plant Biotechnol J. 2016 Nov 22;15(5):568–80. doi: 10.1111/pbi.12654 (PMC5399008; doi:10.1111/pbi.12654)
Supplement: Supplementary file 6 — Table S2 Analysis of yield parameters of WT and RAG2‐RNAi T1 lines. [file PBI-15-568-s002.doc]

**Supplemental Table 1. Analysis of yield parameters of WT and *RAG2-*OX T_1_** lines.

| Plants | Relative expression | Seed set rate (%) | 1000-grain weight (g) | Grain area (mm^2^) |
| --- | --- | --- | --- | --- |
| WT | 1 | 84.34±2.35 | 25.64±1.25 | 8.196±1.013 |
| O2-3 | 1.13 | 82.45±3.44 | 27.34±1.08^＊＊^ | 8.519±1.146^＊^ |
| O2-7 | 1.21 | 80.24±5.93^＊^ | 26.97±2.14^＊＊^ | 9.586±1.279^＊＊^ |
| **O2-15 (OX-1)** | **1.47** | **84.53±4.37** | **27.53±1.42**^＊＊^ | **10.295±1.239**^＊＊^ |
| O2-19 | 2.03 | 76.64±7.18^＊^ | 27.61±0.95^＊＊^ | 10.124±1.386^＊＊^ |
| O2-22 | 1.58 | 83.47±3.53 | 26.78±1.32^＊^ | 10.046±1.266^＊＊^ |
| **O5-5 (OX-2)** | **2.31** | **86.46±4.77** | **27.66±1.11**^＊＊^ | **10.507±1.14**^＊＊^ |
| O5-11 | 1.35 | 85.42±3.19 | 28.09±1.27^＊＊^ | 9.137±1.396^＊＊^ |
| O5-14 | 1.68 | 80.26±4.67^＊^ | 26.73±1.56^＊^ | 9.24±1.488^＊＊^ |
| O5-23 | 2.46 | 82.36±5.01 | 27.10±0.88^＊＊^ | 10.24±1.323^＊＊^ |
| O5-26 | 2.45 | 84.31±2.94 | 27.33±1.31^＊＊^ | 10.256±1.282^＊＊^ |
| O9-5 | 1.72 | 80.21±2.76^＊^ | 25.52±2.53 | 9.574±1.026^＊＊^ |
| O9-8 | 1.27 | 84.62±0.68 | 27.30±2.35^＊＊^ | 9.416±1.295^＊＊^ |
| O9-11 | 2.39 | 82.03±2.15 | 27.32±1.63^＊＊^ | 10.281±1.23^＊＊^ |
| **O9-22 (OX-3)** | **3.18** | **84.45±1.38** | **28.61±1.83**^＊＊^ | **10.681±1.474**^＊＊^ |
| O9-27 | 2.42 | 82.58±2.06 | 28.06±2.57^＊＊^ | 9.496±1.142^＊＊^ |

Rows marked with bold font indicated the lines used for further analysis. Grain size (length, width, and area) of nearly 400 grains from each line measured using MRS-9600TFU2L (grain observation instrument).

Data are mean ± SE for three replicates. ^＊^*P* < 0.05, ^＊＊^*P* < 0.01. *P*-values produced by two-tailed Student’s *t*-test.
