# Supplementary material for: Overexpression of the 16‐kDa α‐amylase/trypsin inhibitor RAG2 improves grain yield and quality of rice
Source: Plant Biotechnol J. 2016 Nov 22;15(5):568–80. doi: 10.1111/pbi.12654 (PMC5399008; doi:10.1111/pbi.12654)
Supplement: Supplementary file 7 — Table S3 Primers used for functional analysis of RAG2. [file PBI-15-568-s008.doc]

**Supplemental Table 2. Analysis of yield parameters of WT and *RAG2-*RNAi T_1_ lines.**

| Line | Relative expression | Seed set rate （%） | 1000-grain weight（g） |
| --- | --- | --- | --- |
| WT | 1 | 87.58±2.43 | 26.82±1.22 |
| i2-3 | 0.33 | 64.33±2.43^＊＊^ | 26.54±1.43 |
| i2-6 | 0.39 | 77.36±1.32^＊＊^ | 25.94±1.06^＊^ |
| i2-15 | 0.42 | 79.24±4.28^＊＊^ | 26.16±0.78^＊^ |
| **i2-21 (Ri-2)** | **0.23** | **50.35±3.46**^＊＊^ | **25.45±1.33^＊^** |
| i2-23 | 0.54 | 59.29±2.32^＊＊^ | 25.37±1.47^＊^ |
| i22-5 | 0.66 | 72.58±3.49^＊＊^ | 26.5±0.66 |
| i22-11 | 0.63 | 63.55±2.82^＊＊^ | 26.33±0.96 |
| **i22-12 (Ri-1)** | **0.45** | **67.42±1.38^＊＊^** | **25.10±1.75^＊^** |
| i22-17 | 0.54 | 67.35±2.27^＊＊^ | 26.33±1.14 |
| i22-23 | 0.72 | 70.67±4.02^＊＊^ | 27.10±0.76 |
| i37-3 | 0.22 | 66.32±3.82^＊＊^ | 25.61±1.41^＊^ |
| **i37-9 (Ri-3)** | **0.13** | **57.34±3.47^＊＊^** | **24.85±1.63^＊＊^** |
| i37-12 | 0.32 | 67.52±4.03^＊＊^ | 25.70±1.62 |
| i37-15 | 0.18 | 60.53±1.98^＊＊^ | 26.42±0.59 |
| i37-19 | 0.39 | 64.23±3.04^＊＊^ | 25.88±1.03^＊^ |

Rows marked with bold font indicated the lines used for further analysis. Data are mean ± SE for three replicates. ^＊^*P* < 0.05, ^＊＊^*P* < 0.01. *P*-values produced by two-tailed Student’s *t*-test.
