# Supplementary material for: Overexpression of the 16‐kDa α‐amylase/trypsin inhibitor RAG2 improves grain yield and quality of rice
Source: Plant Biotechnol J. 2016 Nov 22;15(5):568–80. doi: 10.1111/pbi.12654 (PMC5399008; doi:10.1111/pbi.12654)
Supplement: Supplementary file 8 — Table S1 Analysis of yield parameters of WT and RAG2‐OX T1 lines. [file PBI-15-568-s004.doc]

**Supplemental Table 3. Primers used for functional analysis of *RAG2***.

| Name | Forward primer (5'-3') | Reverse primer (5'-3') |
| --- | --- | --- |
| **Primer for cDNA fragment amplification** | | |
| cRAG2 | ATGGCTTCCAACAAGGTAGT | GTGACCAGTTCTCGGGGTCCTA |
| **Primer for RNAi fragment amplification** | | |
| iRAG2 | GGGACTAGTGGTACCACCACCAATAGATGAAGACC | GGGGAGCTCGGATCCTAGATGCCTCCAACCATG |
| **Primer for overexpression fragment amplification** | | |
| OXRAG2 | GGGGGTACCATGGCTTCCAACAAGGTAGT | GGGGGATCCGTGACCAGTTCTCGGGGTCCTA |
| **Primers for DNA blot hybridization** | | |
| G418 | TGGGAGGTCTATATAAGCAGAG | CGTCGCCGTCCAGCTCGACCAG |
| hph | TCCATACAAGCCAACCAC | TGAAAAAGCCTGAACTCAC |
| **Primers for qRT-PCR** | | |
| *RAG2* | GTGTATGACTCTGTGGGG | CAACGAAATATTGCTACC |
| *RAG1* | GTTCTCGGTATTGCTCCTCGT | GGCTGTAGACTTGGTGGTGGT |
| *Ubi* | AACCAGCTGAGGCCCAAGA | ACGATTGATTTAACCAGTCCATGA |
| *GluA* | ACAAAGAGAAGGATGTGCTTAC | ATTCTTTATCCGCATTGCCAAC |
| *GluB* | CAAGACAAACGCTAACGCCTTC | TCGATAATCCTGGGTAGTATTG |
| *GluD* | AAAGACAATTTCGGACCCTACG | TTAGGAACTGGTAACCCCGCTG |
| *Glb-1* | AGGTTCCAGCCGATGTTCC | CATGCTCTCCTCGTAGCTCCTC |
| *RM1* | TTGTGCAGCAACTACAGCTG | CGGAGCAATGTAGTAGTTAG |
| *Prol14* | ACAACTCCAGCAGTTTGGTG | CAAGGGTGGTAATGGTACTG |
| *RP10* | CAGTTGCCAGATGATGCAGAG | TCAACAACAACCACAGGAAGAGA |
| *RAG-1* | GTTCTCGGTATTGCTCCTCGT | GGCTGTAGACTTGGTGGTGGT |
| *GBSS I* | AAGCGCGCCATCAAGGT | GCACATTCTCCCAGTTCTTCG |
| *SS I* | GGAGAATTTTAACCCGTTTGCT | AAGAGGACTTGTGTTCCCTGTATGT |
| *SS IIa* | TGATGCAGTACACTCGCTCTGT | CTCCGGCAATTCCATGTAGG |
| *SS IIIa* | AATGGGTTCAGCTTTGATGGAG | TCTTGCTCCATGACCCTTTTG |
| *OsAGPL2* | AGTTCGATTCAAGACGGATAGC | CGACTTCCACAGGCAGCTTATT |
| *OsAGPS2b* | AACAATCGAAGCGCGAGAAA | GCCTGTAGTTGGCACCCAGA |
| *OsPPDKB* | GGCAAGTTTCTGCCAACCTACT | CACAAATGCCCACCTCTAAATC |
| *Os08g0510400* | CATCGACGTCCTCATCAACAAC | TTGATAAGTGTCTCCCATTCATCCT |
| *Os09g0505300* | TGTCACTTACATTGGTTGGGTACTT | ATGTTGGCACTCTCTTCTGTAGTCA |
| *GS3* | TCCGCTATATATATCTAGCTGG | CAAAGGCAACCAAGTCATTTGA |
| *GS5* | GTGGAGTACCATGGAATGACAATG | TCATCTGCTTGTCGGAAGCTTC |
| *GL3* | TTACATCGTCACCTCCATGATCGC | CCAGATCGTCCGATCAATGGAG |
| *GW2* | AGAACTTCGCCATGGCGCCAAG | CTACAACCATGCCAACCCTTGC |
| *GW8* | AGTTATCTTGCAAGCCAACAAG | GATTTGGTGGTGCGTGTAGTAT |
